# Phosphorylation of Voltage-Dependent Anion Channel by Serine/Threonine Kinases Governs Its Interaction with Tubulin

**DOI:** 10.1371/journal.pone.0025539

**Published:** 2011-10-13

**Authors:** Kely L. Sheldon, Eduardo N. Maldonado, John J. Lemasters, Tatiana K. Rostovtseva, Sergey M. Bezrukov

**Affiliations:** 1 Laboratory of Physical and Structural Biology, Program in Physical Biology, Eunice Kennedy Shriver National Institute of Child Health and Human Development, National Institutes of Health, Bethesda, Maryland, United States of America; 2 W. Harry Feinstone Department of Molecular Microbiology and Immunology, Johns Hopkins University Bloomberg School of Public Health, Baltimore, Maryland, United States of America; 3 Medical University of South Carolina, Charleston, South Carolina, United States of America; Hertie Institute for Clinical Brain Research and German Center for Neurodegenerative Diseases, Germany

## Abstract

Tubulin was recently found to be a uniquely potent regulator of the voltage-dependent anion channel (VDAC), the most abundant channel of the mitochondrial outer membrane, which constitutes a major pathway for ATP/ADP and other metabolites across this membrane. Dimeric tubulin induces reversible blockage of VDAC reconstituted into a planar lipid membrane and dramatically reduces respiration of isolated mitochondria. Here we show that VDAC phosphorylation is an important determinant of its interaction with dimeric tubulin. We demonstrate that *in vitro* phosphorylation of VDAC by either glycogen synthase kinase-3β (GSK3β) or cAMP-dependent protein kinase A (PKA), increases the on-rate of tubulin binding to the reconstituted channel by orders of magnitude, but only for tubulin at the *cis* side of the membrane. This and the fact the basic properties of VDAC, such as single-channel conductance and selectivity, remained unaltered by phosphorylation allowed us to suggest the phosphorylation regions positioned on the cytosolic loops of VDAC and establish channel orientation in our reconstitution experiments. Experiments on human hepatoma cells HepG2 support our conjecture that VDAC permeability for the mitochondrial respiratory substrates is regulated by dimeric tubulin and channel phosphorylation. Treatment of HepG2 cells with colchicine prevents microtubule polymerization, thus increasing dimeric tubulin availability in the cytosol. Accordingly, this leads to a decrease of mitochondrial potential measured by assessing mitochondrial tetramethylrhodamine methyester uptake with confocal microscopy. Inhibition of PKA activity blocks and reverses mitochondrial depolarization induced by colchicine. Our findings suggest a novel functional link between serine/threonine kinase signaling pathways, mitochondrial respiration, and the highly dynamic microtubule network which is characteristic of cancerogenesis and cell proliferation.

## Introduction

The role of mitochondria in energy production, calcium signaling, and promoting apoptotic signals is well established. There is also emerging evidence of the involvement of mitochondria in multiple other cell signaling pathways with the intimate and dynamic relationship between mitochondria function and cytoskeleton organization and microtubule (MT) network remodeling [1], [2], [3]. The mitochondrial outer membrane (MOM) is the interface between the mitochondria and the cytosol, which serves as a “check point” for the fluxes of metabolites and energy exchange between the mitochondria and other cellular compartments and organelles. A significant portion of the MOM control functions is realized through the voltage dependent anion channel (VDAC) that constitutes a major pathway for ATP/ADP and other mitochondrial metabolites across MOM [4], [5], [6], [7], [8]. Any imbalance in this exchange leads to an essential disturbance of cell metabolism, especially in the processes of development and apoptosis that require extensive mitochondria participation.

Previously, we demonstrated that tubulin induces reversible blockage of VDAC reconstituted into planar lipid membrane and dramatically reduces respiration of isolated mitochondria [9], [10], [11]. Tubulin, the subunit of MT, is a heterodimer composed of α- and β-tubulin subunits. In the presence of tubulin, VDAC conductance fluctuates between the open and tubulin-blocked states. The residual conductance of tubulin-blocked state is relatively high and, in 1 M KCl, is ∼40% of the open state conductance but with the opposite selectivity. At salt conditions close to physiological, in 150 mM vs. 50 mM gradient of KCl, the tubulin-blocked state of VDAC favors cations with an anion-to-cation permeability ratio of 1∶4. This should be compared with the anion selectivity of the VDAC open state with a ratio of 7∶3 [12]. Most importantly, the tubulin-blocked state is virtually impermeable for ATP [12].

We suggested a model in which the negatively charged C-terminal tail (CTT) of tubulin permeates into the channel lumen, interacting with VDAC with high specificity and partially blocking channel conductance for small ions [9], [10]. We also showed that dimeric tubulin reduces respiration of mitochondria isolated from heart and brain [10], [11]. Tubulin increased apparent *K_m_* for ADP, thus dramatically decreasing the availability of ADP to adenine nucleotide translocase. It was concluded that by blocking VDAC permeability for the respiratory substrates tubulin may selectively regulate metabolic fluxes between mitochondria and the cytosol and, therefore, control mitochondrial respiration.

Protein phosphorylation is a key element of the complex network of regulatory and signaling pathways. It is commonly known as an on/off mechanism for numerous cellular processes. Therefore, it is reasonable to expect that phosphorylation of VDAC by the cytosolic kinases might also be involved in regulation of VDAC permeability by tubulin. It was recently shown that VDAC could be phosphorylated by glycogen synthase kinase-3β (GSK3β), and that this phosphorylation is modulated by Akt-dependent inactivation of GSK3β [13], [14]. When hearts were treated with a GSK3β inhibitor in *in vivo* experiments, phosphorylation of VDAC was reduced [13].

Cyclic AMP-dependent protein kinase (PKA) is another serine-threonine kinase with established key roles in cell growth and differentiation. It is also one of the targets in cancer therapy. Phosphorylation of cytoplasmic substrates mediated by PKA is crucial for multiple cell functions, including metabolism, differentiation, synaptic transmission, ion channel activity, and growth and development [15]. PKA was shown lately to co-localize at mitochondria through interaction with the so-called A-kinase anchor proteins and to regulate mitochondrial respiration and dynamics [16]. The local activation of PKA leads to efficient phosphorylation of several mitochondrial substrates [17], suggesting VDAC as a potential PKA target. Indeed, it was reported that VDAC could be phosphorylated by PKA *in vitro* [18].

Here, using mammalian VDAC reconstituted into a planar lipid membrane, we show that VDAC-tubulin interaction appears to be extremely sensitive to the state of VDAC phosphorylation. VDAC phosphorylation *in vitro* by either GSK3β or PKA increased the on-rate of tubulin binding by up to two orders of magnitude. At the same time, the basic properties of VDAC, such as single-channel conductance and selectivity, remained unaltered by phosphorylation. This, and the finding that VDAC phosphorylation increases tubulin binding at the *cis* side of the channel only, suggest that protein regions that are phosphorylated are presumably positioned on the cytosolic loops of VDAC. Modulation of mitochondrial potential by colchicine and PKA inhibitor H89 in human hepatoma HepG2 cells support our conclusion that VDAC phosphorylation by serine/threonine kinases could regulate mitochondrial respiration by affecting VDAC blockage by tubulin.

## Results

### VDAC-tubulin binding strongly depends on VDAC phosphorylation

**Figure 1:**
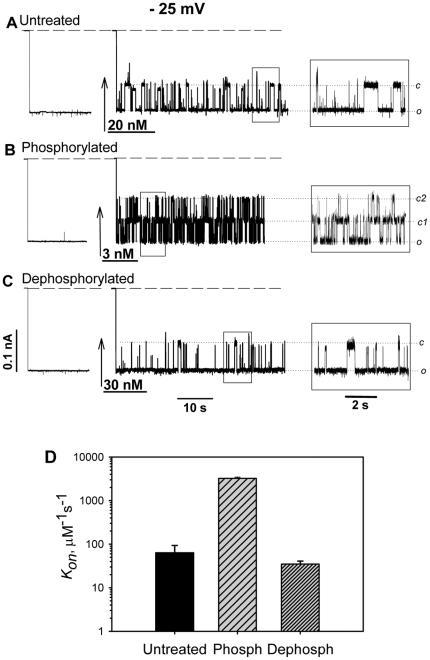
VDAC phosphorylation enhances tubulin-induced VDAC blockage. Records of ion current through two channels in 3 independent experiments after reconstitution of untreated VDAC (A), VDAC phosphorylated with PKA (B), and dephosphorylated with PP2A (C). Current traces on the left in A, B, and C were obtained before tubulin addition in each experiment. Amount of tubulin added is indicated on the traces. While it required 20 nM of tubulin in the *cis* side to induce a moderate blockage of untreated VDAC (A), only 3 nM of tubulin were enough to trigger multiple closure events of phosphorylated VDAC (B). Reversible steps through level c1 to low-conducting level c2 (*inset*) correspond to overlaping closure events when both channels are blocked simultaneously. In contrast, 30 nM were necessary for a moderate blockage of dephosphorylated VDAC (C). Note that only one channel at a time was blocked in traces (A) and (C) (also seen in the *insets*). The applied voltage was −25 mV. Here and elsewhere, dashed lines indicate zero-current levels; the medium consisted of 1 M KCl buffered with 5 mM HEPES at pH 7.4; bilayer membranes were formed from diphytanoyl phosphatidylcholine. Current records were filtered by using averaging times of 10 ms, except for 1 ms in the *insets*. (D) The on-rate constants, *k_on_*, of tubulin binding to untreated, phosphorylated with PKA, and dephosphorylated with PP2A VDAC obtained from kinetic analysis of the experiments illustrated by traces (A–C). Data are mean values obtained in the same experiments at different tubulin concentrations ±S.E.

To study effects of phosphorylation, VDAC isolated from mouse liver mitochondria was incubated with GSK3β or PKA in MgATP cocktail for 1 hour at room temperature to ensure maximum phosphorylation and then reconstituted into planar lipid membranes and tested for its interaction with tubulin. We found that phosphorylation of VDAC by both kinases remarkably enhanced VDAC blockage by tubulin. The representative traces of ion currents through the channels in Fig. 1A–C show that addition of tubulin induces reversible well-resolved fast events of channel closure. The traces of ion currents in the absence of tubulin are shown on the left. The frequency of blockage events strongly depends on VDAC pretreatment with kinase or phosphatase. Addition of 3 nM tubulin to the *cis* side of the membrane induced strong blockage of phosphorylated VDAC at −25 mV (Fig. 1B). The increased sensitivity of VDAC to tubulin is manifested by the events of simultaneous blockage of the two phosphorylated channels that is seen as the transient current reduction to the level designated as *c2*. This should be compared with the behavior of the untreated VDAC (Fig. 1A) where 20 nM of tubulin did not induce events of simultaneous blockage. Furthermore, when VDAC was dephosphorylated with PP2A, 30 nM of tubulin caused even less channel blockage (Fig. 1C) than for the untreated VDAC. Control experiments with VDAC pretreated in MgATP cocktail without kinases yielded no difference in interaction with tubulin in comparison with untreated VDAC (data are not shown).

To quantify the VDAC-tubulin interaction, we performed kinetic analysis of the current blockage events. We first analyzed the distribution of times between blockage events, when the channel stays open, *τ_on_*, and found that it is reasonably well described by a single exponential fitting (Supplemental Fig. S1). Thus, VDAC-tubulin interaction can be adequately represented by a simple binding reaction with the on-rate constant, *k_on_*, which is the inverse average *τ_on_* normalized by tubulin concentration. At −25 mV of the applied voltage the tubulin binding on-rate increased by about two orders of magnitude after phosphorylation of VDAC by PKA. Correspondingly, dephosphorylation of VDAC with PP2A caused a two-fold decrease of *k_on_* (Fig. 1D and Supplemental Fig. S2A).

### Phosphorylated VDAC acquires a pronounced asymmetry

**Figure 2:**
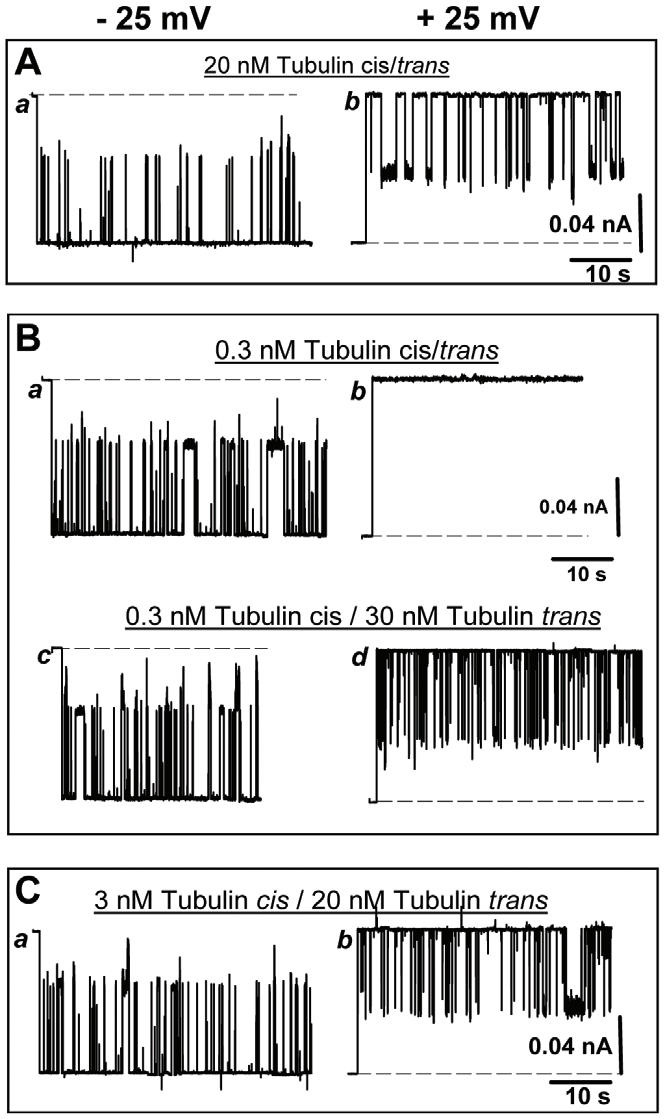
Phosphorylated VDAC is highly asymmetrical in respect to tubulin blockage. (A) For untreated VDAC, 20 nM of tubulin symmetrically added to both sides of the membrane induce nearly symmetrical blockage at negative (*trace a*) and positive (*trace b*) polarities of the applied voltage. (B) Phosphorylation with PKA induces strong asymmetry of blockage. At 0.3 nM of tubulin symmetrically added to the both sides of the membrane, the phosphorylated VDAC is blocked by tubulin at −25 mV (*trace a*) but not at +25 mV (*trace b*). The comparable blockage at +25 mV requires 30 nM of tubulin added to the *trans* side (*trace d)*). (C) VDAC phosphorylated with GSK3β is also asymmetrically blocked by tubulin. Added to the *cis* side 3 nM of tubulin induce about the same frequency of closure events at −25 mV (*trace a*) as 20 nM of tubulin added to the *trans* side at +25 mV (*trace b*).

Another remarkable effect of VDAC phosphorylation is a distinct asymmetry of VDAC blockage by tubulin. Figure 2A shows that untreated VDAC is blocked almost symmetrically at both polarities when tubulin is added symmetrically to the membrane. VDAC phosphorylation with PKA or GSK3β changes this behavior drastically. Representative current traces through a single phosphorylated VDAC are given in Fig. 2B, C. In the case of PKA, with 0.3 nM of tubulin added symmetrically to the membrane (Fig. 2B, *traces a, b*), vigorous VDAC blockage was observed only at negative polarities of the applied voltage (that is, when electric potential at the *cis* side of the membrane is more negative than at the *trans* side). To achieve a similar level of VDAC blockage at the opposite, positive potentials, the concentration of tubulin in the *trans* side had to be increased hundred times up to 30 nM (Fig. 2B, *traces c, d*). Thus, the sensitivity of VDAC to tubulin was increased by two orders of magnitude, but only for VDAC-tubulin interaction at the *cis* side of the channel. The interaction from the *trans* side was virtually unchanged by phosphorylation with PKA.

Similar asymmetry was induced by phosphorylation of VDAC with GSK3β. As shown in Fig. 2C, 3 nM of tubulin in the *cis* side and 20 nM tubulin in the *trans* side induced comparable blockage. When more than one channel was inserted, the asymmetry in tubulin binding to phosphorylated VDAC was maintained (Supplemental Fig. S3).

**Figure 3:**
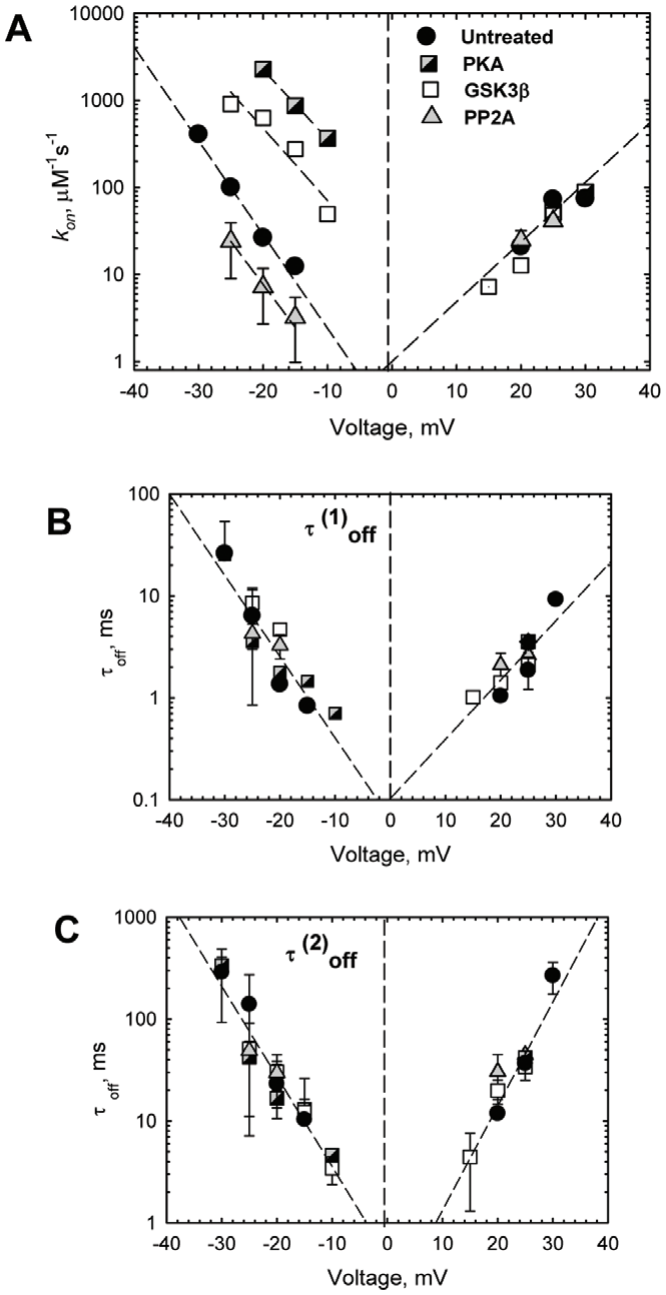
Tubulin binding strongly depends on VDAC phosphorylation but only for tubulin at the *cis* side of the channel. (A) Voltage dependence of *k_on_* at *cis* and *trans* side additions of tubulin. The lines are exponential fits to *k_on_* = *k_0_*exp(n|V|F/RT), where |V| is the modulus of applied voltage and F, R, and T have their usual meaning, with n = 5.4±0.6. Both residence times of VDAC-tubulin binding, *τ* ^(1)^_off_ (B) and *τ* ^(2)^_off_ (C), do not show dependence on the state of VDAC phosphorylation. To reduce the variability of kinetic parameters between different VDAC preparations, all data have been obtained on the same VDAC sample phosphorylated with PKA or GSK3β, or dephosphorylated with PP2A, as indicated in panel (A). Each time value presents the characteristic time of 9 different log probability fitting procedures ±S.E. If error bar is not seen, it is less then the size of the symbol.

Fig. 3 shows a strong exponential dependence of on- and off- rates of VDAC-tubulin binding on the applied voltage. There is ∼300-fold difference in *k_on_* values between phosphorylated and dephosphorylated VDAC, but only at negative potentials. At positive potentials which, under conditions of symmetrical tubulin addition, probe VDAC-tubulin interactions from the *trans* side of the channel, all *k_on_* values collapse onto the same line independently of the state of VDAC phosphorylation. It is interesting that in spite of the large, order of magnitude differences in *k_on_* values, the slopes of the on-rate voltage dependences are almost the same for phosphorylated, dephosphorylated, and untreated VDAC (Fig. 3A). The off-rate of the blockage reaction described by at least two distinctly different characteristic times *τ* ^(1)^_off_ and *τ* ^(2)^_off_ [10] was virtually independent of the VDAC phosphorylation state (Fig. 3B,C and Supplemental Fig. S2B).

Not only the times of tubulin residence in the channel, but also two other basic properties of VDAC, single-channel conductance and selectivity, remained practically unaltered by VDAC phosphorylation (Supplemental Table S1) suggesting that the phosphorylation-induced modifications of VDAC take place outside its water-filled pore.

### Detection of VDAC phosphorylation

**Figure 4:**
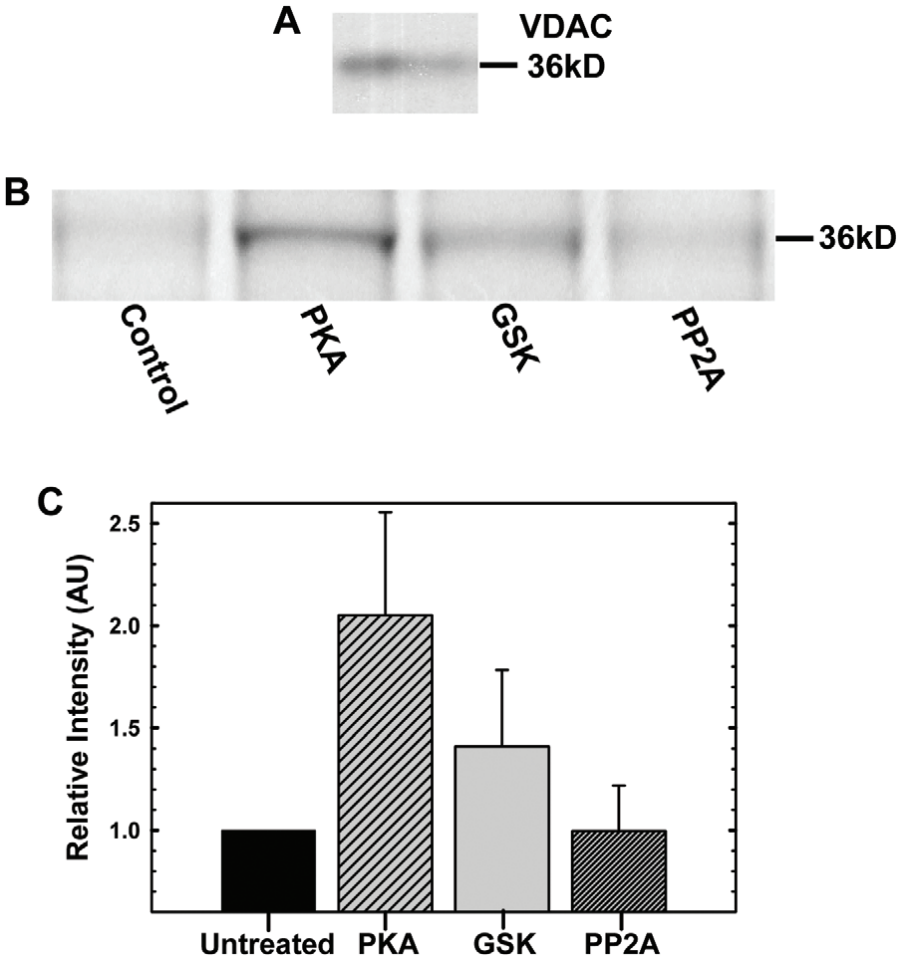
VDAC phosphorylation is detected with phospho-stain. (A), The presence of VDAC in mouse liver mitochondrial membrane fractions was verified by Western blot. (B), Representative Pro-Q Diamond phospho-protein gel stain image of the bands corresponding to VDAC phosphorylated with PKA or GSK3β, or dephosphorylated with PP2A. (C), Relative band intensities normalized versus intensity of VDAC band in the untreated samples in each gel. Data are means of 4 experiments ± S.E.

Phospho-detection was performed on the mouse liver mitochondrial membrane fractions to avoid protein dilution upon column purification and eliminate unwanted intervention of protein detergent with phospho-stain. The presence of VDAC in the mitochondrial fractions was verified by Western blot (Fig. 4A). The representative phospho-protein gel stain image of the bands corresponding to VDAC phosphorylated with PKA or GSK3β, or dephosphorylated with PP2A is shown in Fig. 4B. It is seen that PKA phosphorylates VDAC to a higher extent than GSK3β. This correlates nicely with the higher efficiency of tubulin blockage of VDAC phosphorylated with PKA than VDAC phosphorylated with GSK3β (Fig. 3A). Dephosphorylation with PP2A decreased intensity if only slightly within a standard deviation (Fig. 4B), suggesting that some of the untreated VDAC was already phosphorylated to some extent, and that this level varies significantly among different preparations of mitochondrial fractions. This also correlates with the high variability of *k_on_* values obtained on different VDAC isolation preparations (Supplemental Fig. S2A). It should be noted that in these *in vitro* experiments, dephosphorylation level is compared with untreated VDAC samples and not with samples phosphorylated by kinases. Phosphorylation of the isolated VDAC used in the electrophysiological experiments was verified in the independent experiments (Supplemental Fig. S4). Though the concentration of the isolated VDAC was more than sufficient for membrane reconstitution experiments, it was not high enough to detect VDAC by Coomassie blue and consequently in phospho-stain gels. For that reason, after treatment with kinase or phosphatase VDAC was concentrated by cold chloroform/methanol procedure as described in Supplemental Fig. S4.

### PKA-dependent phosphorylation modulates mitochondrial membrane potential in HepG2 cells

**Figure 5:**
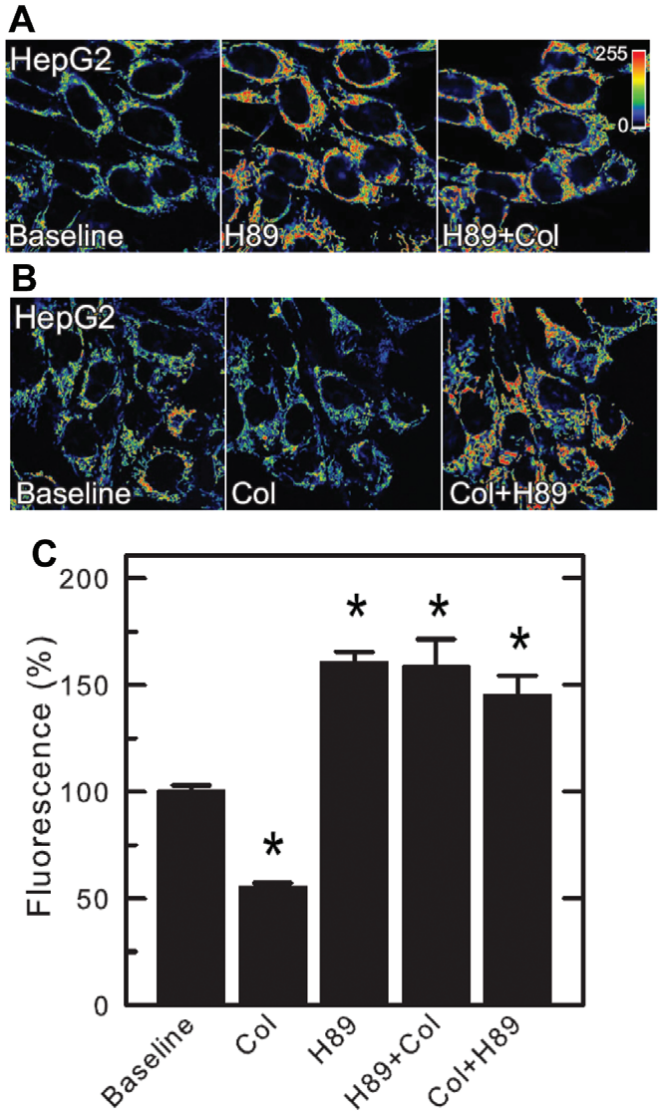
PKA inhibitor H89 blocks and reverses mitochondrial depolarization induced by colchicine in HepG2 cells. Cells in HBSS were loaded with TMRM, as described in Materials and Methods. (A) Baseline cells (*left panel*) were exposed to H89 (1 µM) for 20 min (*center panel*). Colchicine (10 µM) was then added and images were collected after 20 min (*right panel*). (B) After baseline image cells were treated with colchicine (10 µM) for 20 min (*center panel*) before addition of H89 (right panel). (C) TMRM fluorescence after different treatments in relation to untreated cells (baseline). * p<0.05. Experiments were performed in triplicate.

The dramatic effect of VDAC phosphorylation on VDAC-tubulin interaction implies that kinase activation or inhibition should modulate MOM permeability for ADP/ATP and other respiratory substrates and, consequently, mitochondrial inner membrane potential (*ΔΨ*) that is usually called mitochondrial potential. To address this question, we measured *ΔΨ* in HepG2 human hepatoma cells by assessing mitochondrial tetramethylrhodamine methyester (TMRM) uptake using confocal microscopy [19]. It was shown previously that disruption of MT by MT-destabilizers, such as nocodazole and colchicine, decreased mitochondrial membrane potential measured by TMRM uptake by 50–70% [20], whereas stabilization of MT by paclitaxel increased *ΔΨ* by 60%. The amount of free tubulin measured in cancer cells by Western blot rises or drops after colchicine or paclitaxel treatment, respectively [20]. Therefore, the effects of MT-targeting drugs on *ΔΨ* could be explained by modulation of VDAC permeability to mitochondrial metabolites [10] in response to the changes in the concentration of free dimeric tubulin in the cytosol. Since VDAC phosphorylation by PKA increases VDAC blockage by tubulin, we hypothesize that inhibition of PKA should increase MOM permeability to respiratory substrates, consequently increasing *ΔΨ*. In support of this hypothesis, 1 µM of PKA inhibitor, H89, increased mitochondrial TMRM fluorescence by 60% in HepG2 cells (Fig. 5A,C). H89 at this concentration is a specific inhibitor of PKA that does not inhibit other kinases [21].

However, is PKA inhibition/activation coupled to VDAC sensitivity to tubulin, or are there other cellular mechanisms involved? To answer this question we evaluated a possible interrelationship between PKA and free tubulin by treating cells sequentially with H89 and colchicine. Treatment of cells with H89 increased *ΔΨ* and further treatment with colchicine failed to decrease *ΔΨ* (Fig. 5A,C) as occurred after colchicine application in the absence of H89 (Fig. 5B,C). Indeed, cells remained hyperpolarized after colchicine treatment in the presence of H89 (Fig. 5A). Moreover, H89 reversed mitochondria depolarization induced by colchicine and hyperpolarized mitochondria in comparison with the baseline in the absence of both colchicine and H89 (Fig. 5B,C). Thus, H89 inhibits and reverses the effect of colchicine on *ΔΨ*. Supporting these data, another specific PKA inhibitor, a PKI peptide [22], [23] reversed the colchicine-induced mitochondria depolarization similarly to H89 (Supplemental Fig. S5). We conclude that PKA activation is a *sine qua non* condition for tubulin to induce a drop of *ΔΨ*. Therefore, this is in agreement with our findings with the reconstituted channel, where VDAC phosphorylation state determines its blockage by tubulin.

## Discussion

There are emerging data that VDAC is a target of different kinases, in addition to GSK3βand PKA, such as Nek1 [24], PKCε [25], p38 MAP kinase [26], and endostatin reduced hexokinase 2 [27]. A few VDAC post-translational phosphorylation sites have been identified using proteomic approach [28], [29]. This phosphorylation contributes to the VDAC total phosphorylation level and could be involved in different regulatory processes. Indeed, untreated VDAC samples always display a certain level of detectable phosphorylation (Fig. 4). Dephosphorylation of the untreated VDAC with phosphatase PP2A or AP caused some decrease of phosphorylation (Fig. 4, Supplemental Fig. S4) accompanied by mild decrease in *k_on_* of VDAC-tubulin binding (Fig. 1, 3A, Supplemental Fig. S2A). This suggests that post-translational phosphorylation of VDAC also occurs on tubulin-binding sites/domains. Further extensive mass spectrometry analysis is required for unambiguous identification of GSK3β and PKA phosphorylation sites of VDAC.

**Figure 6:**
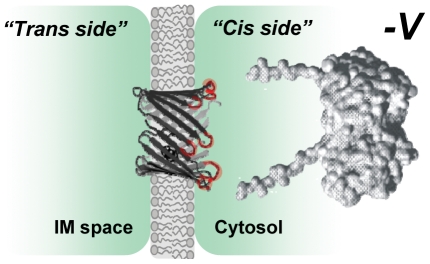
Phosphorylation sites of VDAC are exposed asymmetrically, at the *cis* side of the channel. A scheme of VDAC orientation in reconstitution experiments with the phosphorylation-sensitized tubulin binding sites asymmetrically located on the “*cis*” side of the channel (highlighted in red). Application of negative potentials (−V) drives net negatively charged tubulin in the “cis” side towards the channel. VDAC β-barrel protein (three-dimensional model of mouse VDAC1 is adopted from [30]) is shown embedded in the lipid bilayer with the colored red loops facing the “cis”, or the cytosolic side of the channel. These loops are enriched with eight Thr and five Ser residues and easily accessible for cytosolic kinases. The model of tubulin dimer is adopted from [51].

If some of the residues that face the channel lumen were phosphorylated by one of the kinases, it would introduce an extra negative charge inside the pore and affect channel selectivity making it less anionic. Because selectivity and single-channel conductance were not changed after VDAC phosphorylation with either kinase (Supplemental Table S1), we conclude that phosphorylation occurred on protein region(s) outside of the channel lumen. The loops that connect the β-strands of the VDAC β-barrel are exposed on the membrane interfaces forming channel entrances [30] and they seem to be likely candidates for the region(s) of VDAC phosphorylation and, at the same time, tubulin binding (Fig. 6). According to the 3D VDAC structure [30] the loops on one side of the channel are enriched with five serine and eight threonine residues that all could be potential serine/threonine kinase targets. In a contrast, there is only one serine and four threonines on the loops at the other side of the channel. Interestingly, Thr-51 that was previously suggested [14] as a GSK3β phosphorylation site is located on one of the loops at the channel side enriched with serine/threonine residues. When speculating about potential sites of VDAC phosphorylation by cytosolic kinases we need take into account that these sites should be accessible from the cytosol. Taking together, these considerations and our kinetic data allow us to suggest that PKA and GSK3β phosphorylation regions are positioned on the outside loops at the cytosolic side of the channel. This would explain nicely the strictly asymmetrical effect of VDAC phosphorylation on tubulin binding (Fig. 2, 3A), wherein phosphorylation controls the *cis* side on-rate. For tubulin at the opposite, *trans* side of the channel, *k_on_* does not depend on the VDAC phosphorylation state. Therefore, we conclude that in our reconstitution experiments the loops with phosphorylation regions face the *cis* side of the bilayer, the side of VDAC addition because this side of the channel is sensitized to tubulin interaction by phosphorylation (Fig. 6). This also allows us to suggest that this side corresponds to the cytosolic side of the channel, again taking into account that phosphorylatable sites should be accessible for cytosolic kinases.

Previously, we have shown that the negatively charged CTTs of tubulin are mainly responsible for the VDAC blockage [10]. Tubulin with truncated CTTs, tubulin-S, was unable to block the channel at up to micromolar concentrations. At the same time, the “tailless” tubulin-S generated an excess of high-frequency current noise that is a good indication of some specific interaction of the tubulin globular body with VDAC even without CTT. This initial interaction strongly depends on the state of VDAC phosphorylation, most likely does not involve CTTs, and is able to change the value of the on-rate by orders of magnitude (Fig. 3A). This suggests a two-step binding model where tubulin first binds to the outside loop(s) with phosphorylation sites, and then negatively charged CTTs block the positively charged VDAC pore. Once CTT is inside the pore, it dissociates with the off-rate that is highly voltage dependent but, surprisingly, is not sensitive to the state of VDAC phosphorylation (Fig. 3B,C). Effectively, the consequence of tubulin interaction with the external loops is to increase its concentration at the VDAC pore entrance. There is a close analogy with the effect of adding charge(s) at the entrance of the gramicidin pore [31], [32], which results in the change of local counter-ion concentration.

**Figure 7:**
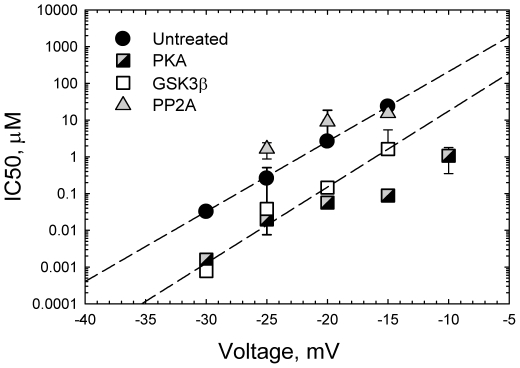
Tubulin inhibitory concentration, *IC50*, spans sub-nanomolar to micromolar range, depending on the applied voltage and VDAC phosphorylation state. The *IC50* values represent averages over datasets obtained in 5–8 experiments with different VDAC samples. The straight interrupted lines are linear regressions through the data of untreated and GSK3β phosphorylated VDAC drawn according to *IC50(V)* = *IC50(0)*exp(nVF/RT), where V is applied voltage, with the “effective gating charge” n = 11.2 and n = 12.2, correspondingly.

Since the *cis* side on-rate is drastically increased by phosphorylation at the approximately conserved off-rate, VDAC phosphorylation state controls the equilibrium constant of the tubulin binding, *K_eq_*. Depending on the applied voltage and the degree of phosphorylation, *K_eq_* values span six orders of magnitude changing from nM^−1^ to mM^−1^. The actual voltage across MOM and its possible variation with the mitochondria state are still debated (see critical discussions in [7], [33]). In spite of the general belief that the potential across MOM is essentially close to zero, the experimental and theoretical estimates for this potential range from 10 mV [33], to 15–20 mV [34], to as high as 46 mV [35]. The last value seems to be overestimation, but if it is true, then for the phosphorylated VDAC, sub-nanomolar tubulin concentrations are sufficient for pronounced blockage. Figure 7 presents the IC50 values for the phosphorylated, dephosphorylated, and untreated VDAC as functions of applied voltage. It is seen that at voltages more negative than −30 mV (not at zero voltages, as was erroneously noted in [10]), the tubulin IC50 is in the nM range.

Under normal physiological conditions, concentrations of dimeric tubulin in the cytosol do not vary dramatically, but in development, under stress and apoptotic stimuli, or in the cancer cells that are characterized by a highly mobile and disintegrated MT network, this situation changes. The equilibrium can be also modified by a family of anticancer drugs, the so-called MT-targeting agents. These agents are known to affect the equilibrium between polymerized and dimeric tubulin [36] leading to mitotic arrest and apoptosis [37], [38]. Indeed, in HepG2 human hepatoma cells, MT polymerization/depolymerization by paclitaxel or colchicine is coupled with changes in inner mitochondrial membrane potential [20] that could be explained by the modulation of VDAC permeability to ATP/ADP and other mitochondrial respiratory substrates by the changing pool of dimeric tubulin in the cytosol. Our results now identify VDAC as a potential new player in the anti-cancer anti-proliferative drug action by linking these drugs to mitochondria bioenergetics. Verification of the role of VDAC blockage by tubulin in the complex network of MT-targeting drug action is a subject of future research.

We show that in HepG2 cells the effect of free cytosolic tubulin, manipulated by colchicine treatment, on mitochondrial membrane potential depends on PKA activity (Fig. 5). PKA activation contributes to the modulation of mitochondrial membrane potential as evidenced by H89 inhibition of colchicine induced depolarization. Experiments with intact cells could be interpreted according to our findings with the reconstituted VDAC, suggesting that tubulin binding to VDAC depends on the state of VDAC phosphorylation and regulates MOM permeability for the respiratory substrates. The discrepancy with the effect of GSK3β inhibition on ATP consumption obtained on isolated cardiac mitochondria [13] is likely due to the difference in VDAC isoforms targeted by GSK3β. Indeed, the VDAC2 isoform was identified as having decreased phosphorylation after GSK3β inhibition [13]. It is believed that VDAC1 is the most abundant isoform in wild type mouse liver mitochondria. Moreover, the phosphorylation effect of GSK3β was confirmed with recombinant VDAC1 (data not shown).

There are other effects of PKA activation/inhibition on mitochondrial functions and biogenesis. Over the last several years it has been recognized that PKA plays a critical role in mammalian mitochondria physiology affecting mitochondrial fussion/fission balance and indirectly regulating autophagy. It was demonstrated that proteins localized on the MOM such as Drp1, a fission protein [39], [40], or Tom70, a component of the translocase outer membrane complex [41], are direct substrates of PKA. The latest results suggest that mitochondrial potential has a role in autophagy regulation [42] and that in yeast the mitochondria function and autophagy could be interdependent [43]. Thus, PKA-mediated control of the mitochondrial potential by VDAC-tubulin interaction is likely to serve, together with other pathways, as a potent coordinator of cellular metabolism. One also should be aware of a possibility of direct action of colchicine on mitochondrial integrity and potential. It was shown that microtubule-targeting drugs could directly transiently hyperpolarize mitochondria and provoke mitochondrial network fragmentation as their early pro-apoptotic activities [44]. Karbowski et al. [3] have shown that depolymerization of MT by nocodazole or colchicine inhibits mitochondrial biogenesis. Therefore, the attenuated effect of colchicine on VDAC-controlled permeability of MOM could be another example of the “multitasking” action of the microtubule-targeting drugs.

Our results also allow us to hypothesize that while VDAC in cancer and normal cells is the same, the state of its phosphorylation could be different. That would lead to a different strength of interaction with tubulin and trigger the consequent modification of mitochondrial potential and mitochondrial respiration. It was recently shown that in primary hepatocytes, in a contrast to cancer cells, paclitaxel and H89 did not cause mitochondrial hyperpolarization [20], whereas MT depolymerization with nocodazole caused a drop of potential similar to cancer cells. However, recent data show a drastically different organization of mitochondria in cardiomyocytes and HL-1 cells of cardiac phenotype. While in normal adult cardiomyocytes mitochondria are arranged into a distinctly regular pattern between MT lattice, they are disorganized into a filamentous mitochondrial network in HL-1 cells [45]. This difference in mitochondria-cytoskeleton intracellular organization is accompanied by the high rate of respiration measured in permeabilized HL-1 cells in comparison with permeabilized cardiomyocytes [1]. Whether such significant differences between mitochondria-cytoskeleton organization, mitochondrial respiration, and response to MT-targeting drugs found in normal and cancer cells are related to VDAC permeability modulation by free tubulin and VDAC phosphorylation state will be answered by future research.

## Materials and Methods

### Protein purification

Frozen mitochondrial membrane fraction of mouse liver was a kind gift of Marco Colombini (University of Maryland, College Park). VDAC was isolated from the membrane fraction by the standard method [46] and then purified on a hydroxyapatite/celite (2∶1) column following the method described in [47]. Bovine brain tubulin was purchased from Cytoskeleton (Denver, CO). Rat brain tubulin was purified according to [48] and was a well-appreciated gift of Dan Sackett (NICHD, NIH). Rabbit skeletal muscle recombinant GSK3β and native alkaline phosphatase (AP) isolated from calf intestine were purchased from Calbiochem, EMD (Gibbstown, NJ). PKA isolated from bovine heart was purchased from Promega (Madison, WI) or Calbiochem, EMD. Human recombinant protein phosphatase 2A (PP2A) was obtained from Cayman Chemical (Ann Arbor, MI).

### Phosphorylation detection

For VDAC phosphorylation 1.0 mg of a wild type mouse mitochondrial membrane fraction was incubated with 1000 units of GSK3β or PKA in 10 µl of kinase buffer (25 mM Hepes at pH 7.4, 150 mM NaCl, 25 mM β-glycerophosphate, 1 mM DTT, and 10 mM MgCl_2_) containing 50 µM MgATP for 30 min at 37°C in the presence of phosphatase inhibitors (Pierce). For dephosphorylation, 1.0 mg of the mitochondrial membrane fraction was incubated with ∼100 units of alkaline phosphatase (AP) or 1 µg of PP2A in 5 µl of Tris HCl at pH 8.0 at 37°C for 30 min. After phosphorylation or dephosphorylation the samples of membranes with kinase in the ATP cocktail were centrifuged for 10 min at 12,000 rpm to collect membrane fraction. The supernatant containing free kinase was discarded and the membranes were processed for running on 4–12% NuPAGE gels (Invitrogen). VDAC bands were verified by Western blotting using a primary anti-VDAC antibody which was a generous gift from Marco Colombini (University of Maryland at College Park). Phosphorylation of VDAC was detected using Pro-Q Diamond phospho-protein stain (Invitrogen). The bands were imaged at 532–560 nm excitation on a Fuji gel scanner, and the difference in staining between control and de- and phosphorylated VDAC was quantified using “Multi Gauge V3.0” software. The VDAC band intensities with gel background subtracted were normalized versus intensity of the untreated samples in each corresponding gel. All gels were analyzed in raw, unmodified state.

For electrophysiological experiments instead of the whole mouse liver mitochondrial fraction, 10 µl of purified VDAC in 2.5% triton x-100 were phosphorylated following the above procedure at 32°C (a duration and temperature of VDAC phosphorylation were optimized experimentally to give a higher yield). After phosphorylation or dephosphorylation the sample of VDAC with kinase in the ATP cocktail was directly used in the channel reconstitution experiments. Phosphorylation of the isolated VDAC was verified as described above and in Supplemental Fig. S4.

### Reconstitution experiments

Planar lipid membranes were formed on 70–80 µm diameter orifices in the 15-µm-thick Teflon partition that separated two chambers as previously described [49]. Lipid monolayers used for membrane formation were made from 5 mg/ml solution of diphytanoyl phosphatidylcholine (DPhPC) (purchased from Avanti Polar Lipids, Inc. Alabaster, AL) in pentane. Aqueous solutions of 1 M KCl were buffered with 5 mM Hepes at pH 7.4. VDAC channel insertion was achieved by adding 0.1–1.5 µl of a 2.5% triton x-100 solution of purified VDAC to the 1.2 ml of the potassium chloride solution in the *cis* compartment while stirring. Potential is defined as positive when it is greater at the side of VDAC addition (*cis*). After VDAC channels were inserted and their parameters were monitored, tubulin was added to one or both sides of the membrane under constant stirring for 2 min. Current recordings were performed as described previously [49] using an Axopatch 200B amplifier (Axon Instruments, Inc., Foster City, CA) in the voltage clamp mode. Single-channel data were filtered by a low-pass 8-pole Butterworth filter (Model 9002, Frequency Devices, Inc., Haverhill, MA) at 15 kHz and directly saved into the computer memory with a sampling frequency of 50 kHz. For data analysis by Clampfit 9.2 a digital 8-pole Bessel low pass filter set at 500 Hz was applied to all records, and then individual events of current blockages were discriminated. Lifetimes were calculated by fitting logarithmic single or double exponentials to logarithmically binned histograms of at least 150 blockage events [50]. Nine different logarithmic probability fits were generated using different fitting procedures and the mean and standard deviation of the fitted time constants were plotted as the lifetime.

### Cell imaging

HepG2 cells (ATCC, Manassas, VA) were grown in Eagle's minimum essential medium supplemented with 10% fetal bovine serum, 100 units/ml penicillin and 100 µg/ml streptomycin in 5% CO_2_/air at 37°C. For confocal microscopy, cells were cultured on glass bottom culture dishes (MatTek, Ashland, MA) for 48 h. Cells in modified Hank's balanced salt solution (HBSS) containing (in mM): NaCl 137, Na_2_HPO_4_ 0.35, KCl 5.4, KH_2_PO_4_ 1, MgSO_4_ 0.81, Ca_2_Cl 0.95, glucose 5.5, NaHCO_3_ 25 and HEPES 20, pH 7.4 were loaded 30 min at 37°C with 200 nM of tetramethylrhodamine methyester (TMRM). After loading and washing, subsequent incubations were performed with 50 nM TMRM to maintain equilibrium distribution of the fluorophore [19]. All microscopy experiments were performed in 5% CO_2_/air at 37°C in modified (HBSS). Colchicine and H89 were purchased from Sigma (St. Louis, MO) and TMRM from Invitrogen (Carlsbad, CA). All other reagents were analytical grade. TMRM-loaded cells were placed on the stage of a Zeiss LSM 510 inverted laser scanning confocal microscope (Thornwood, NY) in humidified 5% CO_2_/air at 37°C. Fluorescence of TMRM was imaged with a 63× 1.4 N.A. planapochromat oil immersion lens using 543-nm HeNe laser (0.5–1.5% full power) excitation, respectively. Emitted red fluorescence of TMRM was detected through a 560-nm long-pass filter and a one Airy unit diameter pinhole. Differences between groups were analyzed by the Student's *t*-test using *p*<0.05 as the criterion of significance. Results were expressed as means ± SEM of three or more experiments.

## Supporting Information

Figure S1 **Times between successive tubulin-induced VDAC blockages, open times, strongly depend on VDAC phosphorylation.** Statistical analysis of the open times, *τ*_on_, in the experiments, examples of which are presented in Fig. 1, was performed by logarithmic exponential fitting. Open-time histograms are satisfactory described by single exponents with characteristic time *τ*_on_ equal to 415, 52, and 519 ms for VDAC untreated, phosphorylated with PKA, and dephosphorylated with PP2A, respectively.
(TIF)

Figure S2 **On-rate of VDAC-tubulin binding strongly depends on the level of VDAC phosphorylation, while residence times remain unaltered.** On-rate, *k_on_,*, (*A*) and two residence times, *τ* ^(1)^_off_ and *τ* ^(2)^_off_, (*B*) are the average values obtained in multiple experiments with VDAC phosphorylated with PKA or GSK3β and dephosphorylated with AP or PP2A in comparison with untreated VDAC. The applied voltage was −20 mV. Experimental conditions were as in Fig. 1. Number of experiments is indicated on the graphs. Note, that different mouse VDAC isolation preparations were used that resulted in high variability of *k_on_* values. Data are means ± S.E. Statistical analysis of *k_on_* values was done using two-tailed t-test (a = 0.05) as a comparison with untreated VDAC samples (P<0.2).
(TIF)

Figure S3 **The phosphorylation-induced asymmetry of blockage is maintained after simultaneous insertion of many channels.** Current records of 5 channels obtained with VDAC phosphorylated with GSK3β. 2 nM of tubulin in the *cis* side induce more frequent closure events at −20 mV applied voltage than 20 nM of tubulin added to the *trans* side at +20 mV. Simultaneous closure of more than one channel at −20 mV is seen. Experimental conditions as in Fig. 2.
(TIF)

Figure S4 **Detection of phosphorylation of isolated VDAC.** (A) Pro-Q Diamond phospho-protein gel stain image (i) of untreated VDAC isolated from mouse liver mitochondria and phosphorylated with PKA or dephosphorylated with AP and subsequent Comassie blue staining of VDAC. To improve Coomassie blue and phospo-stain gels resolution, isolated VDAC was concentrated by cold chloroform/methanol procedure before applying to SDS-PAGE gels and treated to the phosphoprotein gel stain (see Invitrogen protocol). The dry sample was resuspended in SDS sample buffer and run on 4–12% SDS Page Gels (Invitrogen). The gel was treated with Pro-Q Diamond Phospho-protein stain as described in the Methods. The band of auto-phosphorylated PKA (39 kDa) is clearly seen. VDAC band at 36 kDa is seen in Commassie blue gel (ii). (B) Relative band intensities shown in *Inset*, normalized versus untreated VDAC samples (control). Number or repeats 3.
(TIF)

Figure S5 **PKI blocks depolarization induced by colchicine in HepG2 cells.** Cells in HBSS were loaded with TMRM, as described in Materials and Methods. (A), Baseline cells were exposed to colchicine (10 µM) alone for 20 min (*upper panel*) or after treatment with PKI, a PKA peptide inhibitor (20 µM) for 30 min (*bottom panel*). (B), TMRM fluorescence is plotted for the different treatments.* p<0.05 vs other groups.
(TIF)

Table S1 Characteristic channel properties of VDAC remain unaltered after phosphorylation.
(PDF)
