# Supplementary figures and images for: Phosphorylation of Voltage-Dependent Anion Channel by Serine/Threonine Kinases Governs Its Interaction with Tubulin
Source: PLoS One. 2011 Oct 13;6(10):e25539. doi: 10.1371/journal.pone.0025539 (PMC3192757; doi:10.1371/journal.pone.0025539)

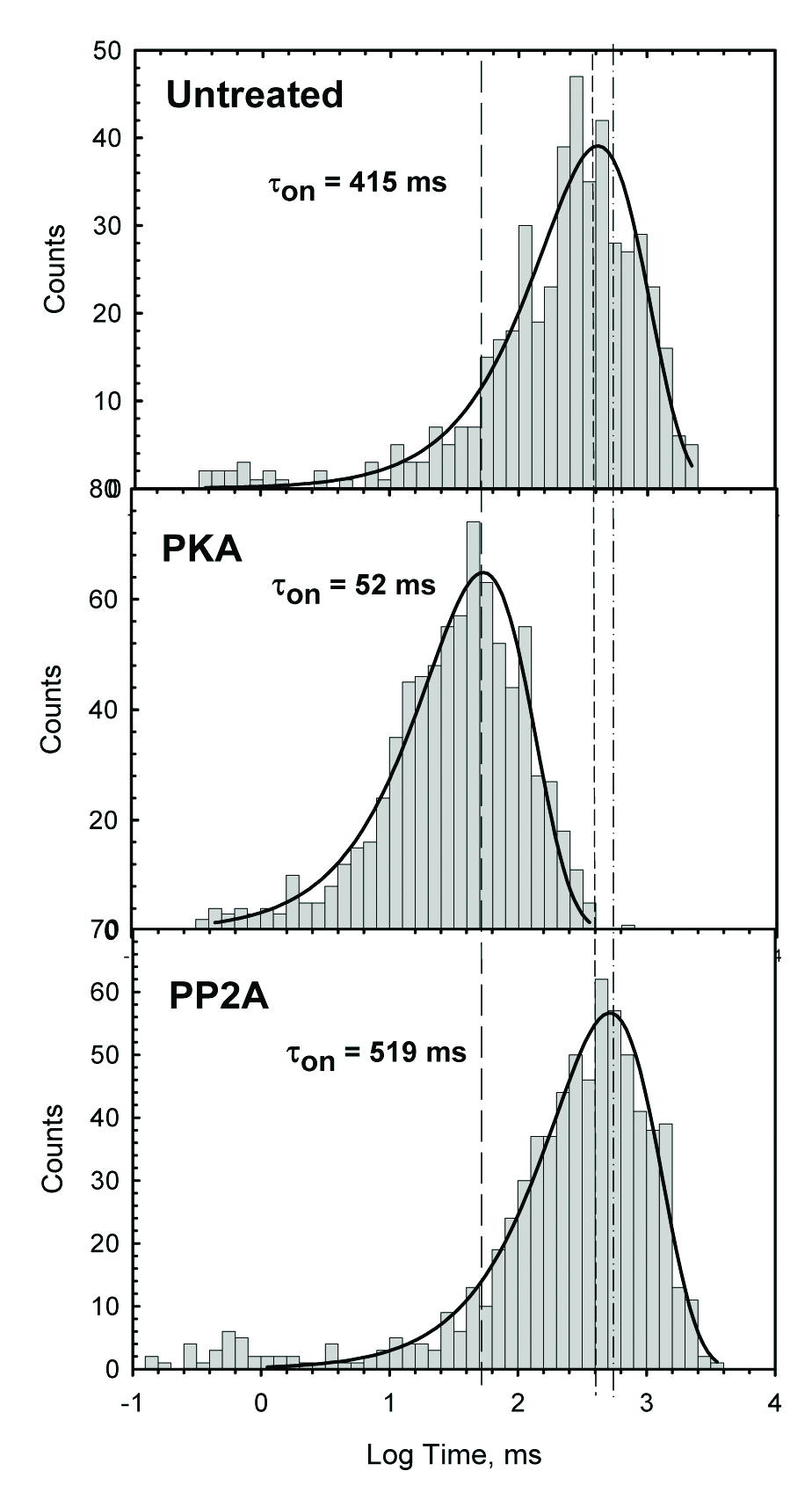

Supplement: Figure S1 — Times between successive tubulin-induced VDAC blockages, open times, strongly depend on VDAC phosphorylation. Statistical analysis of the open times, τ on, in the experiments, examples of which are presented in Fig. 1, was performed by logarithmic exponential fitting. Open-time histograms are satisfactory described by single exponents with characteristic time τ on equal to 415, 52, and 519 ms for VDAC untreated, phosphorylated with PKA, and dephosphorylated with PP2A, respectively. (TIF) [file pone.0025539.s001.tif]

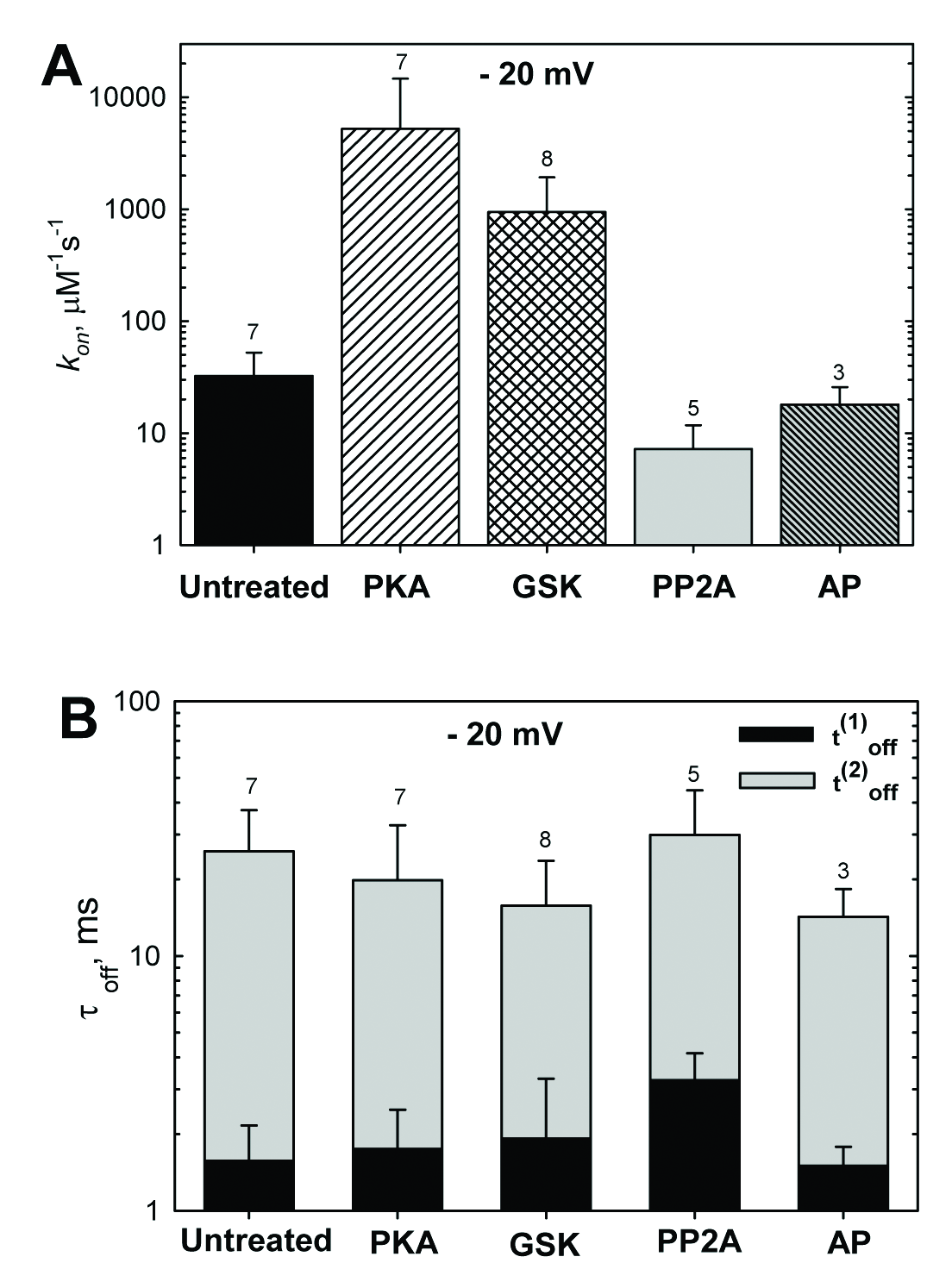

Supplement: Figure S2 — On-rate of VDAC-tubulin binding strongly depends on the level of VDAC phosphorylation, while residence times remain unaltered. On-rate, kon,, (A) and two residence times, τ (1) off and τ (2) off, (B) are the average values obtained in multiple experiments with VDAC phosphorylated with PKA or GSK3β and dephosphorylated with AP or PP2A in comparison with untreated VDAC. The applied voltage was −20 mV. Experimental conditions were as in Fig. 1. Number of experiments is indicated on the graphs. Note, that different mouse VDAC isolation preparations were used that resulted in high variability of kon values. Data are means ± S.E. Statistical analysis of kon values was done using two-tailed t-test (a = 0.05) as a comparison with untreated VDAC samples (P<0.2). (TIF) [file pone.0025539.s002.tif]

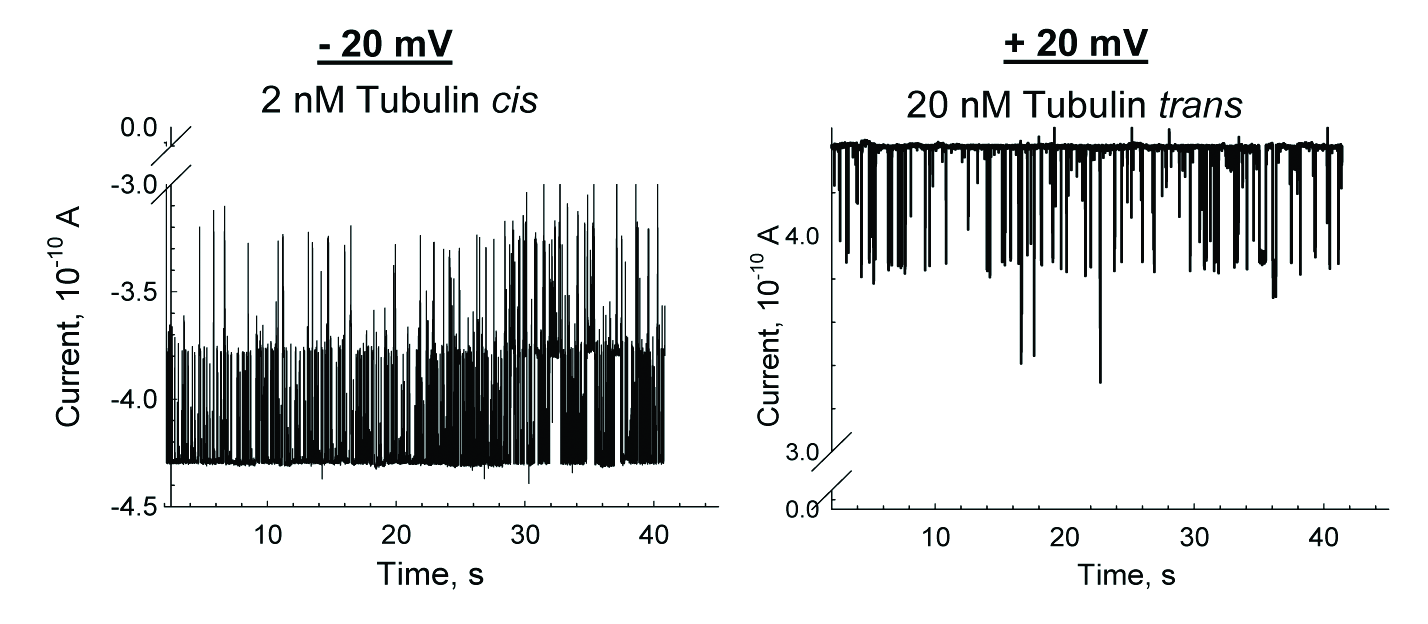

Supplement: Figure S3 — The phosphorylation-induced asymmetry of blockage is maintained after simultaneous insertion of many channels. Current records of 5 channels obtained with VDAC phosphorylated with GSK3β. 2 nM of tubulin in the cis side induce more frequent closure events at −20 mV applied voltage than 20 nM of tubulin added to the trans side at +20 mV. Simultaneous closure of more than one channel at −20 mV is seen. Experimental conditions as in Fig. 2. (TIF) [file pone.0025539.s003.tif]

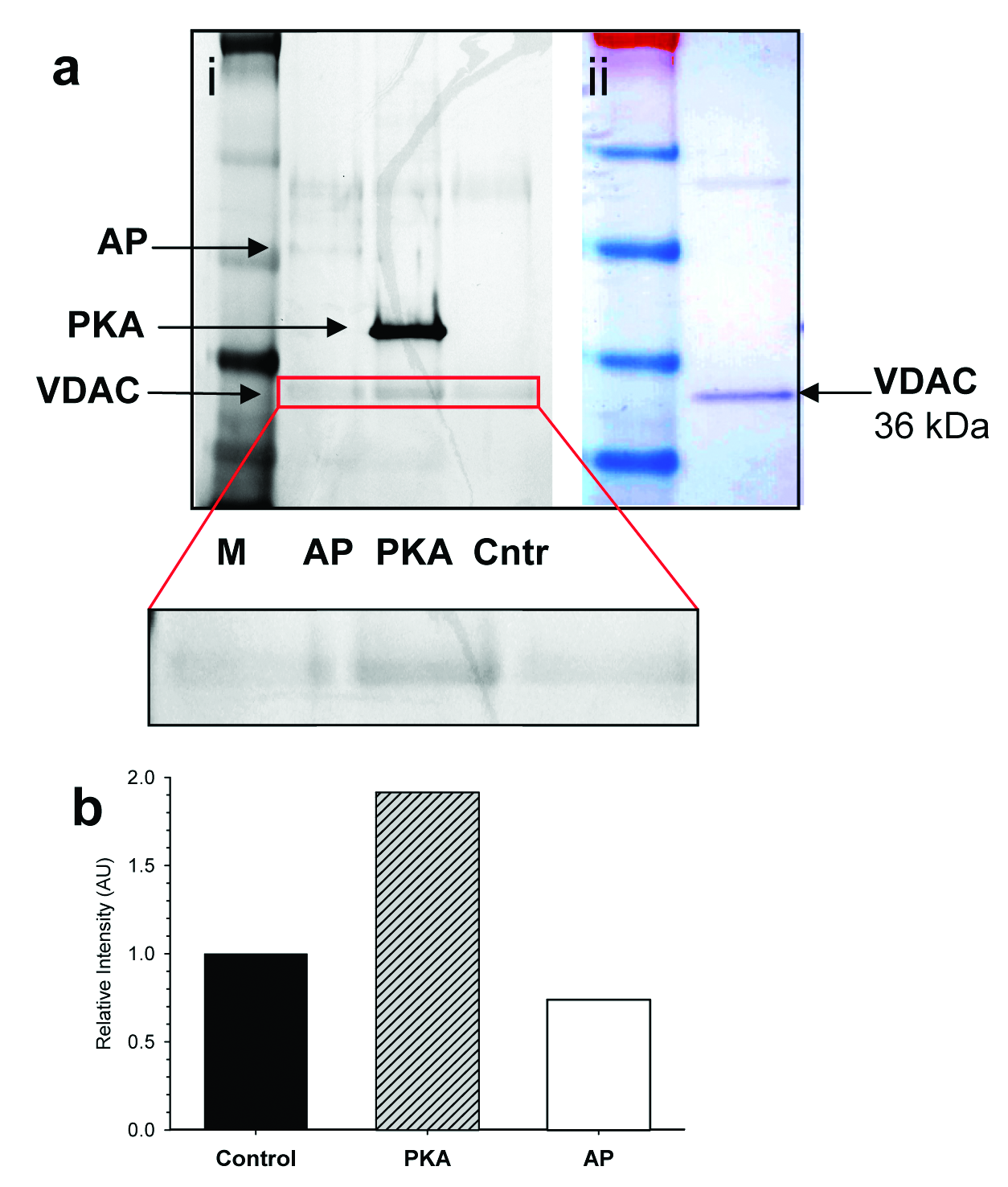

Supplement: Figure S4 — Detection of phosphorylation of isolated VDAC. (A) Pro-Q Diamond phospho-protein gel stain image (i) of untreated VDAC isolated from mouse liver mitochondria and phosphorylated with PKA or dephosphorylated with AP and subsequent Comassie blue staining of VDAC. To improve Coomassie blue and phospo-stain gels resolution, isolated VDAC was concentrated by cold chloroform/methanol procedure before applying to SDS-PAGE gels and treated to the phosphoprotein gel stain (see Invitrogen protocol). The dry sample was resuspended in SDS sample buffer and run on 4–12% SDS Page Gels (Invitrogen). The gel was treated with Pro-Q Diamond Phospho-protein stain as described in the Methods. The band of auto-phosphorylated PKA (39 kDa) is clearly seen. VDAC band at 36 kDa is seen in Commassie blue gel (ii). (B) Relative band intensities shown in Inset, normalized versus untreated VDAC samples (control). Number or repeats 3. (TIF) [file pone.0025539.s004.tif]

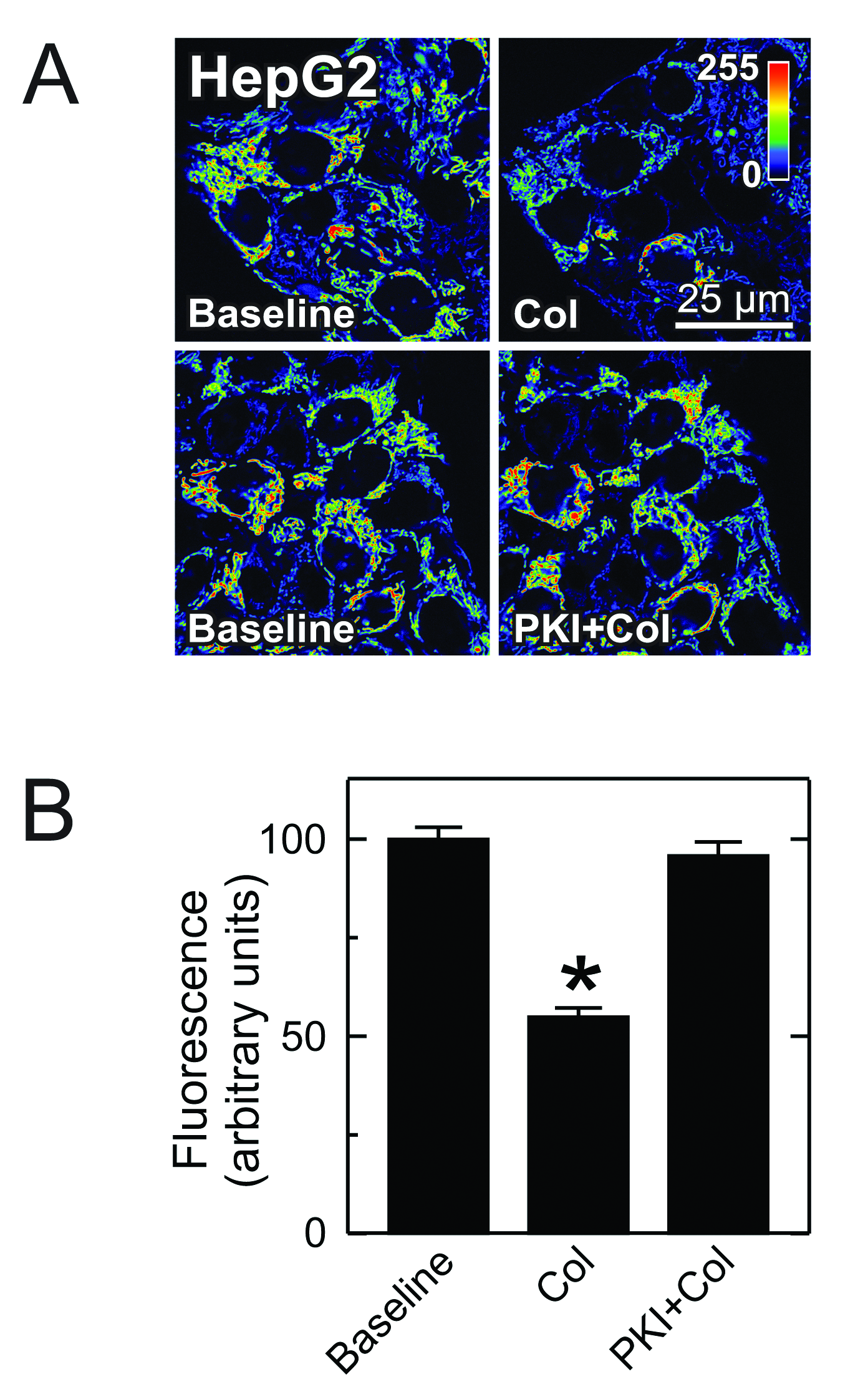

Supplement: Figure S5 — PKI blocks depolarization induced by colchicine in HepG2 cells. Cells in HBSS were loaded with TMRM, as described in Materials and Methods. (A), Baseline cells were exposed to colchicine (10 µM) alone for 20 min (upper panel) or after treatment with PKI, a PKA peptide inhibitor (20 µM) for 30 min (bottom panel). (B), TMRM fluorescence is plotted for the different treatments.* p<0.05 vs other groups. (TIF) [file pone.0025539.s005.tif]
