# Supplementary material for: Phosphorylation of Voltage-Dependent Anion Channel by Serine/Threonine Kinases Governs Its Interaction with Tubulin
Source: PLoS One. 2011 Oct 13;6(10):e25539. doi: 10.1371/journal.pone.0025539 (PMC3192757; doi:10.1371/journal.pone.0025539)
Supplement: Table S1 — Characteristic channel properties of VDAC remain unaltered after phosphorylation. (PDF) [file pone.0025539.s006.pdf]

Supplemental Table S1.
Characteristic channel properties of VDAC remain unaltered after phosphorylation

	Single channel conductance in 1 M KCl, nS	Reversal potential in 1.0/0.2 M KCl, mV	Permeability ratio P_{Cl^-}/P_{K^+}
Untreated VDAC	4.0 ± 0.2 (7)	8.7 ± 0.7 (3)	1.5
Phosph w/ PKA	4.3 ± 0.4 (9)	7.4 ± 1.4 (4)	1.5
Phosph w/GSK3β	4.2 ± 0.2 (11)	7.0 ± 2.0 (4)	1.5
Dephosph w/PP2A	4.2 ± 0.07 (6)	8.7 ± 0.2 (2)	1.5

Each value is a mean of n experiments indicated in parentheses ± S.E.
